# American Medical Association

**Published:** 1869-06

**Authors:** 


### AMERICAN MEDICAL ASSOCIATION.

The American Medical Association met in the hall of the Mechanics' Institute, in the City of New Orleans, May 4th, 1869.

The following is a reliable abstract of its proceedings, corrected from the *New Orleans Times*:—

### PROCEEDINGS OF THE OPENING DAY.

The American Medical Association met in the Mechanics' Institute, at 11 A.M.

The President, Dr. W. O. Baldwin, of Alabama, occupied the Chair, assisted by Vice-Presidents Drs. Geo. Mendenhall, of Ohio, and S. M. Bemis, of Louisiana.

The Permanent-Secretary, Dr. W. B. Atkinson, of Pennsylvania, and Assistant-Secretary, Dr. A. J. Semmes, of Georgia, were present.

The President invited to seats on the platform Drs. Warren Stone and A. Lopez, of New Orleans, and ex-Presidents H. F. Askew, of Delaware, N. S. Davis, of Illinois, and Alden March, of New York.

The session was opened with prayer by Rev. Mr. Gallaher, of New Orleans.

Dr. T. G. Richardson, of Louisiana, Chairman of the Committee of Arrangements, welcomed the delegates to the city in an eloquent address.

He announced that the session would be held from 9 A.M. to 2 P.M., and the Sections would meet at 3 P.M., in the Mechanics' Institute, and in the University.

On motion of Dr. Richardson, the following gentlemen were elected members by invitation:—Drs. Taney, Legare, Anfoux, Tebault, Barnes, of New Orleans, and McFarland, of Mississippi.

The President then delivered the annual address.

On motion of Dr. Askew, of Delaware, it was referred to the Committee of Publication.

Letters were read by the Permanent-Secretary from Drs. S. D. Gross, of Pennsylvania, W. Byard, and W. Canniff, of Canada, and R. A. Kinloch, Chairman of Medical Society of South Carolina, expressing regret at their inability to be present on this occasion.

Reports of special committees were called for.

Disease of Cornea.—No report.

Cultivation of Cinchona Tree, reported progress, and on motion of Dr. Toner, District of Columbia, Dr. T. Antisell, District of Columbia, was added to that committee.

Excision of Joints for Injuries.—No report.

Alcohol and its Relation to Medicine.—Dr. Jno. Bell, Pennsylvania, presented a report, which was referred to the Section of Practice of Medicine.

On the Cryptogamic Origin of Disease, with special reference to Recent Microscopic Investigations on that Subject.—Dr. Edward Curtis, U. S. A., Chairman. Reported, and referred to Section on Meteorology and Epidemics.

On Operations for Hare-lip: Dr. A. Hammer, Missouri, Chairman.—No report.

On Clinical Thermometry in Diphtheria: Dr. Jos. G. Richardson, New York, Chairman.—Discharged at his own request.

On Prophylactics in Zymotic Diseases: Dr. Nelson L. North, New York, Chairman.—Reported and referred to Section on Meteorology and Epidemics.

On Inebriate Asylums: Dr. C. H. Nichols, D. C., Chairman. —No report.

On the influence of the Pneumogastric Nerve on Spasmodic and Rhythmical Movements of the Lungs: Dr. Thomas Antisell, D. C., Chairman.—No report.

To Examine into the Present Plan of Organization and Management of the United States Marine Hospitals: Dr. D. W. Bliss, D. C., Chairman.—No report.

On the Utilization of Sewerage: Dr. Stephen Smith, New York, Chairman.—No report.

On the Influence of Quarantine in Preventing the Introduction of Disease into the Ports of the United States: Dr. Elisha Harris, New York, Chairman.—No report.

On Nurse-Training Institutions: Dr. Samuel D. Gross, Pennsylvania, Chairman.—Reported, and referred to Section on Practical Medicine.

On Commissioners to Aid in Trials Involving Scientific Testimony: Dr. John Ordronaux, New York, Chairman.—Reported, and referred to Section on Medical Jurisprudence.

On Annual Medical Register: Dr. John H. Packard, Pennsylvania, Chairman.—Reported progress, and, on motion of Dr. Mussey, of Ohio, it was—

*Resolved*, That each State Medical Society be requested to prepare an Annual Register of all the regular practitioners of medicine in their respective States, giving the name of the colleges in which they may have graduated, and date of diploma or license.

On Devising a Plan for the Relief of Widows and Orphans of Medical Men: Dr. John H. Griscom, New York, Chairman. —Reported, which was referred to the Committee of Publication.

On Veterinary Colleges: Dr. Thomas Antisell, District of Columbia, Chairman.—Reported progress and was continued.

On Specialities in Medicine, and the Propriety of Specialists Advertising: Dr. E. Lloyd Howard, Maryland, Chairman.—Reported, and was, on motion, made the special order for Wednesday, at 12 M.

On Library of American Medical Works: Dr. J. M. Toner, D. C., Chairman.—Reported, and was, on motion of Dr. Davis, made special order for Wednesday, at 1 P.M.

On Vaccination: Dr. Henry A. Martin, Massachusetts, Chairman.—No report.

On the Decomposition of Urea in Uræmic Poisoning: Dr. H. R. Noel, Maryland, Chairman.—No report.

On the Best Method of Treatment for the Different Forms of Cleft Palate: Dr. J. R. Whitehead, New York, Chairman. —Reported and referred to Section on Surgery.

On Rank of Medical Men in the Navy: Dr. N. S. Davis, Illinois, Chairman, announced that their last year's report was final, and committee was discharged.

The report on Medical Ethics by Dr. D. Francis Condie, Pennsylvania, Chairman, was read by Dr. Davis, and adopted.

On American Medical Necrology: Dr. C. C. Cox, Maryland, Chairman.—Reported progress, and was continued on motion of Dr. Davis. Dr. Cox was authorized to fill all vacancies on his committee.

Voluntary communications were presented by Dr. Jos. Jones, of Louisiana, on Mollities Ossuim; and referred to Section on Surgery.

On Cases of Lead Palsy from Use of Cosmetics. By Dr. L. Sayre, of New York. Referred to Section on Hygiene.

On the Physiology and Chemistry of Longevity. By Dr. Cutler, of Mississippi. Referred to Section on Hygiene.

On the Protective and Preventive Uses of Quinine. By Dr.

S. Rogers, of New York. Referred to Section on Practical Medicine.

On the Tongue in Malarious Diseases. By Dr. Osborn, of Alabama. Referred to Section on Practical Medicine.

On the Warm Cerebro-Spinal Bath in the Treatment of Congenital Apnœa, and on a New Method of Artificial Respiration. By E. D. McDaniel, of Alabama. Referred to Section on Practical Medicine.

Reports on Climatology and Epidemics were received from Drs. Thoms, of New York; T. J. Heard, of Texas; F. W. Hatch, of California; E. A. Hildreth, of West Virginia; which were referred to the Section on Climatology and Epidemics.

Reports of progress were received from Drs. Hamill, of Illinois; A. Sager, of Michigan; Compson, of Mississippi; and Pimm, of Louisiana.

On the motion of Dr. Davis, the Report on the Revision of the Plan of Organization was made the special order for Wednesday, at 10 A.M.

Papers relative to Medical Education were read, and referred, on motion of Dr. Davis, to a special committee of five, to be appointed by the President.

The President appointed Drs. Davis, Illinois; P. F. Eve, Tennessee; E. S. Gaillard, Kentucky; E. Lee Jones, New York; and J. K. Bartlett, Wisconsin.

On motion, adjourned until Wednesday, at 9 A.M.

SECOND DAY'S PROCEEDINGS.

At 9 A.M., Dr. W. O. Baldwin, the President, in the Chair, called the meeting to order. The reading of the minutes having been dispensed with, Dr. Richardson, the Chairman of the Committee of Arrangements, presented the names of the following candidates for admission, by invitation, to the Association, who were duly elected:—

Dr. Jas. E. Morris, New Iberia, La.; Drs. Wm. H. Watkins, John M. Cullen, Charles H. Kelly, S. R. Hurd, C. J. Bickham, P. B. McKelvey, Wm. G. Austin, J. Bensadon, O. Anfoux, H. D. Schmidt, Fr. Loeber, S. A. Smith, of New Orleans; L. L.

Henry, Henderson McFarland, J. S. Bacon, of Mississippi; Dr. C. Tucker, of Danville, Kentucky; and Drs. Florence O'Donnoghue, and John F. Randolph, of the U. S. A.

A paper on "Canula and the New Mode of Applying Ligatures," was submitted by Dr. P. F. Eve, Tennessee, and was referred to the Section on Surgery.

Dr. J. M. Bush, of Ky., offered the following resolution:—

*Resolved*, That a committee of five members be appointed by the Chair, to take into consideration the subjects alluded to in the President's address, and report at this meeting.

This resolution having been adopted, the President selected as members of the committee, Dr. Parvin, of Indiana, Chairman; Dr. Toner, of the District of Columbia; Dr. Pollock, of Pennsylvania; Dr. Welch, of Texas; Dr. Seely, of Alabama.

Dr. McPheeters, of Missouri, offered a communication from the Medical Association of that State, in reference to Medical Education.

On motion of Dr. Toner, District of Columbia, it was referred to the Special Committee on that subject.

Dr. Eve offered the minutes of the Medical Society of Tennessee, which was similarly referred.

Dr. Gaillard, of Kentucky, offered the following preamble and resolutions which were referred to the same committee:—

*Whereas*, The medical teachers of America have, after a trial of twenty-two years, failed to meet satisfactorily and efficiently the requirements of the great body of the profession in regard to medical education; and

*Whereas*, The condition of the profession is yearly becoming more deplorable, on account of the antagonistic and objectionable policy of medical schools, in making the amount of fees charged, rather than a successful teaching, the basis of competition; and

*Whereas*, To obtain professionally competent graduates, sound and efficient teachers are indispensably necessary; and

*Whereas*, Such teachers, to be found throughout the country, cannot be induced to leave their homes, without the assurance of competent remuneration; and

*Whereas*, Such remuneration can only be obtained by adequate fees charged, unless by a system of low fees the *number* of students be relied upon to make up the inevitable pecuniary deficiency; and

*Whereas*, Reliance upon numbers of students for this purpose deplorably crowds the already overcrowded professional field, diminishing thereby individual income, judgment, experience, and skill, thereby compelling practitioners to resort to other avocations as a source of supplemental income; and

*Whereas*, This devotion to other pursuits destroys opportunities for study and improvement, degrading thereby the status and standard of American physicians; and

*Whereas*, The schools of New England, New York, Pennsylvania, Maryland, Virginia, South Carolina, Georgia, Alabama, Texas, Tennessee, and District of Columbia now charge comparatively remunerative fees; and

*Whereas*, the low system of fees is charged only in a few of the Middle States, and can with advantage be made to conform to the rate of fees charged elsewhere; and

*Whereas*, It is as unethical for colleges to underbid each other pecuniarily or for practitioners to do so:

*Resolved*, That hereafter no medical school in this country, other than those fully endowed, be entitled to representation in this Association, if the amount charged by such schools for a single course of regular lectures be less than $140,00.

*Resolved*, That all schools charging less than this sum are earnestly requested by this Association to advance their rate of fees to the amount mentioned.

The report of Dr. Lee, of New York, the delegate to the Association of Superintendents of Insane Asylums, was offered, and referred to the Section on Psychology.

The report of Dr. Gross, of Pennsylvania, delegate to Foreign Medical Association, was presented, together with the letter to Dr. Ehrenberg, was read, and referred to the Committee of Publication.

The time having arrived for consideration of the Revision of Plan of Organization, it was, on motion, taken up.

On motion of Dr. Hibberd, the following amendment to the constitution was adopted:—

Add to Article VII. the following:—"*Provided*, However, that when an amendment is properly under consideration, and an amendment is offered thereto, germain to the subject, it shall be in order, and, if adopted, shall have the same standing and force as if proposed at the preceding meeting of the Association."

On motion, the following amendments to the constitution, submitted last year, were adopted:—

II. MEMBERS.

In this section, second paragraph, fourth line, insert after the words "United States" the words "from the army and navy."

In fifth paragraph, third line, insert after the word "member" the words "or whose name shall have been, for non-payment of dues, dropped from the rolls of the same." In fifth line, same paragraph, after the word "sentence," read "or disability." In sixth line, after the word "society," add the following:—"Or shall have paid up all arrears of membership; nor shall any person, not a member and supporter of a local medical society, where such a one exists, be eligible to membership in the American Medical Association."

In seventh paragraph, fifth line, strike out the remainder of sentence after the word "by," and insert the words "at least three of the members present, or three of the absent permanent members." In ninth line, after the word "delegates," add the words "except the right to vote."

In eighth paragraph, fifth line, add after the word "delegates," the words "and comply with the requirements of the by-laws of the Association.

In ninth paragraph, third line, insert after the word "must" the words "exhibit his credentials to the proper committee."

III. MEETINGS.

In first paragraph, third line, strike out after the word "shall" the words "never be the same for any two years in succession, and shall."

After the ninth paragraph insert the following new sentence: —"Corresponding members shall consist of such medical gentlemen, eminent in their profession, residing out of the United States, as the Association shall, from time to time, elect."

IV. OFFICERS.

In first paragraph, third line, after the word "Treasurer," insert the words "and Librarian." In second line, after the word "Secretary," strike out the article "and."

The following amendment was, after much discussion, unanimously rejected:—In the third line, after the word "Librarian," insert the following new sentence:—The President shall be nominated and ballotted for in open convention, and shall be elected only from those who have attended at least five annual meetings of the Association; and if, on the first ballot, no person receives a majority of the votes cast, the second ballot shall be confined to the three highest on the list; should no choice be then made, the candidate lowest on the list shall then be dropped. In the event of a tie on the third or succeeding ballot, the President shall decide by a casting vote."

After eighth paragraph, insert a new paragraph as follows: —"The Librarian shall receive and preserve all the property in books, pamphlets, journals, and manuscripts presented to or acquired by the Association, record their title in a book prepared for the purpose, acknowledge the receipt of the same, and he shall also be a member of the Committee of Publication."

V. STANDING COMMITTEES.

In second paragraph, second line, insert after the word "members" the words "of whom the Assistant-Secretary shall be one."

In third paragraph, first line, strike out the word "and." In second line, after the word "Treasurer," read "and Librarian."

VI. FUNDS AND APPROPRIATIONS.

In first paragraph, fifth line, insert after the word "the" the words "delegates and permanent." In the same line, strike out the word "individual."

VII. PROVISION FOR AMENDMENT.

In the first paragraph, fourth line, strike out the word "members," and insert the word "delegates."

BY-LAWS—III. STANDING COMMITTEES.

In second paragraph, ninth line, strike out all after the word "resolution."

In third paragraph, fourth line, after the word "receive" insert the word "original." In same line, after the word "any," insert the word "medical."

In third paragraph, eleventh line, strike out the word "volunteer," and insert the word "original."

In sixth paragraph, second line, after the word "State," insert "and Territory." In fourth line, strike out the words "our country," and insert the words "their respective States and Territories." In same line, strike out all after the word "and," and insert the words "shall transmit them to the Chairman of this committee, on or before the first of April of each and every year."

V. ASSESSMENTS.

In fourth line, strike out the word "the," and in same line all after the word "expenses" to the end of the sentence.

In second paragraph, first line, strike out all after the word "invitation," and insert the following sentences:—"Permanent members not in attendance will transmit their dues to the Treasurer. Any permanent member who shall fail to pay his annual dues for three successive years, unless absent from the country, shall be dropped from the roll of permanent members."

On motion of Dr. Davis, of Illinois, the amendment was amended as follows:—"After having been notified by the Secretary of the forfeiture of their membership."

The amendment was adopted as amended.

The following were adopted as read:—

VII. DELEGATES TO FOREIGN MEDICAL SOCIETIES.

In first paragraph, fourth line, after the word "Europe," insert the words "or other foreign countries."

X. OF THE PREVIOUS QUESTION.

When the previous question is demanded, it shall take at least twenty members to second it; and when the main question is put under force of the previous question and negatived, the question shall remain under consideration, the same as if the previous question had not been enforced.

A recess was taken to allow the selection of members of the Committee of Nominations.

On reassembling, the Permanent-Secretary announced the following as the Committee on Nominations:—

New York—J. C. Smith.
Delaware—H. F. Askew.
Pennsylvania—A. M. Pollack.
Kentucky—H. M. Skillman.
Tennessee—J. B. Lindsley.
Mississippi—W. Y. Gadbury.
Alabama—J. Cochran.
Ohio—John Townsend.
Indiana—B. S. Woodworth.
Illinois—T. D. Fitch.
Wisconsin—H. Van Dusen.
Missouri—J. S. Moore.
Michigan—J. B. White.
Georgia—R. D. Arnold.
Louisiana—S. Logan.
Texas—S. M. Welch.
Minnesota—C. N. Hewitt.
Arkansas—R. G. Jennings.
West Virginia—W. J. Bates.
Rhode Island—G. L. Collins.
District of Columbia—L. W. Ritchie.
United States Army—J. J. Woodward.
United States Navy—F. E. Potter.

Dr. Chaille, of Louisiana, submitted a proposition for a common medical nomenclature in the United States, taking as a model an official publication on the subject by the Royal College of Physicians of London, and offered the following resolutions, which were adopted:—

*Resolved*, That a committee of five be appointed by the President, to report, as soon as practicable, to the present session of this Association, the following:—

1. The propriety of adopting and using its influence to have adopted, by the entire medical profession in the United States, the provisional "Nomenclature of Diseases of the Royal College of Physicians."

2. On the practibility of having this nomenclature published in such manner as may render it easily and cheaply accessible to every member of the profession.

3. To recommend such other practical measures for the action of this Association as may be necessary to introduce this nomenclature into official (military, naval, etc.) and general use.

The Chair appointed the following gentlemen as the committee:—Drs. Woodward, U. S. A., Heustis, of Alabama, F. G. Smith, of Pennsylvania, and Chaille, of Louisiana.

Dr. Cochran, of Alabama, offered the following amendments, which were laid over under the rules:—

1. Section 2, paragraph 1—That the clause "as members by invitation" be stricken out.

2. That the second paragraph be stricken out.

3. That of paragraph fourth, all shall be stricken out except the first sentence.

4. That paragraph seven, of "members by invitation," be stricken out.

The reports of the Committee of Publication and the Treasurer were read, accepted, and referred to the Committee of Publication.

On motion the Committee on Nominations were permitted to retire for consultation.

The special order for 12 o'clock being the Report on Specialists, it was read by the Secretary; and, on motion of Dr. Sayre, the resolutions were adopted, and the report referred to the Committee of Publication.

Dr. L. P. Yandell, Jr., of Kentucky, offered the following, which was adopted:—

*Resolved*, That private handbills addressed to members of the medical profession, or by cards in medical journals, calling the attention of professional brethren to themselves as specialists, be declared in violation of the Code of Ethics of the American Medical Association.

The special order for 1 o'clock being the Report on American Medical Library, Dr. Toner, Chairman read the report.

After some discussion, on motion of Dr. Hibberd, of Indiana, the report was accepted.

Dr. Davis, of Illinois, offered the following resolutions:—

*Resolved*, That the proposition of the Librarian of the Congressional Library be accepted.

*Resolved*, That a committee of one be appointed, residing at Washington, to render the Librarian of Congress such assistance as the interests of the Association may require.

Adopted.

Report on Medical Education presented, and, on motion of Dr. Hibberd, of Indiana, it was made the special order for 10 o'clock to-morrow.

Report on Prize Essays reported:—

The undersigned, appointed Committee on Prize Essays, at the session of 1868, respectfully report:—

They have received but two essays—one upon "The Physiological Effects and Therapeutical Uses of Atropia and its Salts;" the other upon "Quinine as a Therapeutic Agent." They agree to present both of these essays to the Association, and to recommend the award of a prize of one hundred dollars to each of them.

S. M. Bemiss, Chairman,
C. Beard, M.D.,
Joseph T. Scott, M.D.,
S. A. Smith.

The Secretary broke the seals and announced that Dr. S. S. Herrick, of New Orleans, was the author of the paper on quinine, and Dr. Roberts Bartholow, of Cincinnati, was the author of that on atropia.

On motion of Dr. Davis, the Sections were authorized to meet at 3½ P.M. in place of 3.

Remarks upon certain points referring to success in the operation of vesico-vaginal fistula, by M. Schuppert, M.D., of New Orleans, Louisiana.

Referred to Section on Obstetrics.

Dr. Booth, of Mississippi, offered the following preamble and resolution:—

*Resolved*, That the proper construction of Art. 4, Sec. 1, Code of Ethics, A. M. A., having been called for, relative to consultation with irregular practitioners, who are graduates of regular schools;

*Resolved*, That said Art. 4, Sec. 1, Code of Ethics, A. M. A., excludes all such practitioners from recognition by the regular profession.

On motion, the Convention adjourned until Thursday, at 9 A.M.

THIRD DAY'S PROCEEDINGS.

Dr. Baldwin, President, in the Chair. Dr. S. M. Bemiss, and Dr. Mendenhall, Vice-Presidents.

Reading of the minutes was dispensed with.

Dr. Parvin reported from the Committee on President's Address, as follows:—

We cannot refrain, before entering upon the consideration of the plan recommended by the President for the improvement of medical education, gladly expressing our high appreciation of the general tone of his address, of the broad and catholic spirit which pervades it, finding expression in earnest and eloquent words; in brief, we believe the address worthy the perusal of every member of the profession, in that it was worthy the memorable occasion, and is worthy the annals of medicine.

On the other hand, we cannot refrain—with sadness be it said—from acknowledging the truth of the terrible allegations made against the present condition of medical education, and the little success attending the efforts for improvements in such connection, made during a score of years.

The special recommendation made by the President is in these words:—

"I would advise that we appoint a committee of our wisest and best men, to digest a plan for one or more national medical schools, and to memorialize Congress on behalf of the enterprise. Let the plan embrace as a basis the features presented by the Cincinnati Convention of Teachers; let these schools or universities confer such distinctions and privileges as will be proportionate to the superiority they demand, and such as will make the attainment of their diploma an object of the ambition of those who engage in the study of medicine; let the choice be open to all aspirants, and the appointment or election of professors so guarded as to secure the very highest talent, the most profound learning, with the most fully demonstrated capacity for teaching. Make the salaries of the professors large, and not to depend upon the number of students; and let the Federal Government assume a proper share of the expenses incurred."

Your committee express their hearty approval of this general plan, but suggest that the effort at first should be for the establishment of but a single school, as more feasible; and beside, one such institution would be a model which other medical colleges might in time be induced to imitate in extent, duration, and thoroughness of teaching, in rigidness of requirements for the degree of M.D.

We likewise desire to say that when the details of this general plan are thrown into form, there should be the amplest security against the places and the power of such a medical college as designed ever falling into the hands of politicians, or the proteges of politicians. Medicine is higher than politics—broader than political creeds and party platforms.

In conclusion, your committee reiterate the recommendation of the President as to the appointment of a committee for the special purpose referred to.

Drs. Parvin, Welch, Seely, Toner, and Pollock, committee.

Dr. Hibberd moved acceptance, was adopted, and, on the proposition of Dr. Davis, the committee was ordered to consist of five. The President appointed Dr. N. S. Davis, of Illinois; Dr. F. G. Smith, of Pennsylvania; Dr. D. H. Storer, of Massachusetts; Dr. E. S. Gaillard, of Kentucky; and Dr. Joseph Jones, of Louisiana.

On motion of Dr. Davis, Dr. W. O. Baldwin was added to the Committee.

Dr. Palmer, of Michigan, submitted the following amendments to the by-laws, which were adopted:—

Amend Section 11 of by-laws, by inserting in place of the clause after "6 Psychology," "each section, etc.," these words: —"The President and Secretary of the several Sections shall, like other officers of the Association, be nominated by the Special Committee of one member from each State represented at the meeting, and elected by a vote on general ticket. They shall hold their office until the close of the proper business of the annual meeting next succeeding their election, and until their successors are appointed.

Modify next paragraph appropriate to the several sections; in order to secure consideration and action, must be sent to the Secretary of the appropriate Section, at least one month before the meeting which is to act upon them. It shall be the duty of the Secretary to whom such papers are sent to examine them with care, and, with the advice of his Section, to determine the time and order of their presentation, and give due notice of the same; and after their full examination and discussion by the Section, they shall be sent to the Permanent-Secretary of the Association.

Papers presented directly to the Association, and other matters, may, at the discretion of the Association, be referred to the various sections for their consideration and report.

The President appointed as delegates to the British Medical Association:—

Dr. N. Pinckney, U. S. N.; R. R. McIlvain, Ohio; J. F. Hibberd, Indiana; B. Lindsley, D. C.; G. C. Blackman, Ohio.

To the Canadian Medical Association:—

Dr. Alden March, Albany, N. Y.

To the Committee on Ethics was appointed:—

Dr. Sayre, N. Y.; Toner, D. C.; Askew, Del.; Arnold, Ga.; McCluskey, Ala.

Dr. Hibberd presented handbill put out by Dr. J. B. Buchtil, of Terre Haute, Indiana, and charged irregular practice in this conduct. Paper was read and referred to the Committee on Ethics.

Dr. Davis presented the following from the Association of American Medical Editors:—

To the American Medical Association:—I have been instructed to announce to your honorable body that those members of your Association in attendance on this annual meeting who are editors of medical periodicals, after proper consultation, have effected a permanent organization with the title of "The Association of American Medical Editors." The objects of this organization are the cultivation of friendly relations, mutual assistance, community of effort and views, where possible, in a system of receiving foreign exchanges, and sending our own journals abroad, concert of action in support of improvements in the present system of medical education, and of a higher standard of preliminary attainments for those who propose to enter upon the study of medicine, in proposing laws for the proper registration of births, marriages, and deaths, in collecting the names of all the regular practitioners in the several states, and in promoting generally the value and efficiency of our periodical medical literature. The Association thus formed is to hold its annual sessions on the day preceding the annual meetings of this body, and in the same localities. Dr. Mitchell, of New Orleans, is the Permanent-Secretary, and Dr. J. B. Lindsley, of Nashville, Tenn., the Assistant-Secretary. Congratulating your honorable body on the establishment of another organized power within the ranks of your noble profession, I remain, yours most truly,

N. S. Davis, *Editor*,
*President of Association of American Medical Editors.*

Referred to Committee on Publications.

The Secretary presented a paper from Dr. Walsh, of Georgia, referring to the action of the Georgia Medical Society in his case. Referred to Committee on Ethics.

Dr. Gaillard, of Kentucky, explained the Kentucky troubles. No action.

Dr. Parvin read report of Dr. J. C. Reeves, on Medical Education, which had been made special order for 10 o'clock A.M. Adopted, and referred to Committee on Publications.

Dr. McPheeters, of Missouri, offered a resolution that no speech should be longer than ten minutes. Adopted.

The Committee on Nominations—Dr. J. J. Woodward, U. S. N., President—reported the following names:—

REPORT OF THE NOMINATING COMMITTEE.

NEW ORLEANS, LA., *May 6th, 1869.*

The Committee on Nominations unanimously report as follows:—

*For President*—George Mendenhall, Ohio.

*For Vice-Presidents*—Warren Stone, La.; Lewis A. Sayre, N.Y.; F. Gurney Smith, Penn.; John S. Moore, Mo.

*For Assistant-Secretary*—Wm. Lee, D. C.

*For Treasurer*—Caspar Wister, Penn.

*For Librarian*—Robert Reyburn, D. C.

*Committee of Arrangements*—Thomas Antisell, Chairman; Robert Reyburn, C. M. Ford, L. W. Ritchie, W. J. C. Duhamel, D. R. Hayner, C. F. Nally.

*Committee of Publication*—F. Gurney Smith, Pa., Chairman; W. B. Atkinson, Pa.; A. J. Semmes, Ga.; Robt. Reyburn, D. C.; Caspar Wister, Pa.; H. F. Askew, Del.; Wm. Maybury, Pa.

*Committee on Medical Literature*—J. J. Woodward, U. S. A., Chairman; W. H. Anderson, Ala.; Theophilus Parvin, Ind.; Hosmer A. Johnson, Ill.; C. W. Parsons, R. I.

*Committee on Prize Essays*—Grafton Tyler, D. C., Chairman; N. R. Lincoln, D. C.; N. R. Smith, Md.; G. W. Miltenberger, Md.; W. R. Dunbar, Md.

*Committee on Epidemics*—Add the following to fill vacancies: —J. K. Bartlett, Wis.; J. B. Jackson, Ky.

*Committee on Education*—T. G. Richardson, La., Chairman; E. W. Jenks, Mich.; E. S. Gaillard, Ky.; W. M. McPheeters, Mo.

Time for meeting, in Washington, first Tuesday in May, 1870.

J. J. WOODWARD, U. S. A., *Chairman.*

The report was unanimously adopted.

Dr. Herrick, Louisiana, offered amendment to Code of Ethics, on the duties of physicians to each other and the profession at large.

ART. I. DUTIES FOR THE SUPPORT OF PROFESSIONAL CHARACTER—PROPOSED AMENDMENT—ADDITIONAL SECTION.

SECTION 5. The spirit of trade and of gain from merchandise should by all means be dissociated from the practice of a liberal profession; and it is important that practitioners should not allow their pecuniary interests to compromise their duties to their patients. Therefore, in cities and other communities where the services of competent apothecaries can conveniently be obtained, physicians should resign to them the whole business and profits of dispensing medicines.

Laid over to next yearly session.

Dr. Davis offered a report on various propositions and communications from medical societies, which was adopted and referred to Committee on Publication.

Dr. Davis offered the following:—

*Resolved*, That a special committee of three be appointed by the President, to present copies of the resolutions adopted, to the several State Medical Societies, at as early a period as possible. Adopted.

The President appointed as such Committee Drs. N. S. Davis, of Ill., J. S. Wetherly, of Ala., and J. M. Toner, of D. C.

Dr. Chaille, of Louisiana, Chairman of committee, presented a report on Medical Nomenclature, which was received and adopted, and referred to Committee on Publication.

The Committee on the Nomenclature of Diseases have the honor to report that it has examined the "Provisional Nomenclature of the Royal College of Physicians," of London, and is of the opinion that it is desirable for this Association to recommend and adopt the same for general use in this country, with such modifications as, on deliberate consideration, may appear to be necessary. The following resolutions are, therefore, submitted:—

1. *Resolved*, that a special committee of fifteen be appointed by the President to take this subject into deliberate consideration, and to report at the next annual session what alterations, if any, are necessary to adapt the proposed nomenclature to general use in the United States.

2. That this committee be authorized to fill up any vacancies which may occur upon it.

3. That the Committee on Publications be authorized to publish, for general distribution, one thousand copies of the English and Latin portions of this nomenclature, under the designation of the "Proposed Nomenclature," prefacing the same with such remarks as may be deemed necessary to secure the criticism and coöperation of as large a number of the American medical men as practicable.

4. That the committee hereby appointed be directed to draw the attention of the Surgeon-General of the army, of the Chief of Bureau of Medicine and Surgery of the navy, and of the Superintendent of the Census, to the question of their official adoption of the proposed nomenclature; to invite them to appoint whom they see fit to represent them on this committee; and to solicit such coöperation as may be necessary to accomplish the purpose desired, *viz.:* the final adoption of such nomenclature and classification as will receive the conjoint approval of the official medical bureaus of the Government and of the general profession.

*Committee*—S. E. Chaille, Louisiana; J. J. Woodward, United States Army; A. B. Palmer, Michigan; F. G. Smith, Pennsylvania; J. F. Heustis, Alabama.

The following committee of fifteen was appointed:—

Francis G. Smith, Chairman; J. J. Woodward, U. S. A.; R. F. Michel, Alabama; A. B. Palmer, Michigan; S. E. Chaille, Louisiana; L. P. Yandell, Jr., Kentucky; Austin Flint, New York; Alonzo Clark, New York; Geo. B. Wood, Pennsylvania; S. H. Dickson, Pennsylvania; E. Jarvis, Massachusetts; Theo. Parvin, Indiana; W. M. McPheeters, Missouri; E. M. Snow, Rhode Island; N. Pinkney, U. S. N.

Capt. Neal, of the steamer Richmond, invited the members to take an excursion at 5 P.M.

On motion of Dr. Garrish, of New York, thanks were tendered to Capt. Neal.

Dr. Garrish, New York, offered a vote of thanks to the N. O., J., and G. N. R. R., for agreement to pass members back free. Adopted.

Dr. Gaillard, Kentucky, offered the following, with preliminary remarks:—

*Resolved*, That the adoption of a uniform rate of collegiate fees—$120 being the minimum—be accepted as the sentiment and desire of this Association.

Dr. Logan, of Alabama, moved to amend by inserting $140.

After considerable discussion, the fees were placed at $120.

Special Committee on the Relative Advantages of Syme's and Pirogoff's mode of Amputating at the Ankle.—Dr. G. A. Otis, U. S. A., Chairman; Dr. J. D. Holloway, of Louisville, Kentucky.

Proposed by J. J. Woodward. Approved.

Dr. Bemiss presented from Dr. John Waters, of St. Louis, Mo., a paper on "The Doctrines of Force—Physical and Vital."

Dr. A. M. Pollock, of Pennsylvania, presented this amendment to the constitution:—

*Resolved*, That all County Medical Societies shall be required to elect a committee annually, whose duty it shall be to examine all applicants for admission as students under the tuition of its members; and that no member of any county medical society shall receive any such applicant, until such applicant shall present a certificate from said committee, testifying that he has a good English education, and a sufficient knowledge of Greek and Latin to enable him to pursue his studies with advantage.

Laid over under the rules.

Dr. Toner, D. C., moved that a committee on variola be appointed—Dr. Jos. Jones, Chairman. Adopted.

Dr. Pinckney, U. S. N., made statements concerning relative grades of rank. The paper was ordered to be spread on the minutes.

Association adjourned, to meet at 9 o'clock A.M., Friday, May 7th.

FOURTH DAY'S PROCEEDINGS.

The Association met at 9 o'clock, Dr. Baldwin in the Chair.

Reading of the minutes omitted.

In yesterday's report the paragraph which defines the rates of fees in medical colleges is corrected so as to read that "the adoption of a uniform rate of collegiate fees—120 dollars being the minimum—be accepted as the sentiment and desire of the Association."

Dr. Joseph Jones, Louisiana, presented a number of specimens of pathology, anatomy, and natural history. The explanations were very interesting, and received with applause.

On Motion of Dr. Garrish, Kentucky, the thanks of the Association were tendered to Dr. Jones.

The following delegates were appointed to foreign Medical Societies:—

To the Canadian Medical Association:—H. F. Askew, Del.; R. Miller, Alabama; J. M. Bush, Kentucky; N. S. Davis, Ill.; Riggs, Alabama.

To the International Medical Association:—Dr. W. J. C. Duhamel, District of Columbia; Jos. Berney, Alabama; E. L. Jones, New York; B. F. Dawson, New York; Joseph Jones, New Orleans.

To the British Medical Association:—F. A. Ross, Alabama.

Delegate to Association of Superintendent of Insane:—Robert Reyburn, District of Columbia.

On motion of Dr. F. G. Smith, of Pennsylvania, the following resolution was unanimously adopted by a vote of the members present, standing as a mark of respect:—

*Resolved*, That the thanks of this "Association" are justly due and are hereby tendered to the President for the uniform kindness and courtesy with which he has presided over its deliberations, and to the Committee of Arrangements, the physicians and citizens of New Orleans, for the generous hospitality and fraternal kindness with which we have been received and treated during our sojourn in their city, with the assurance that the memories of this visit will always be among the brightest and most enduring of our lives.

*Resolved*, That we also present our thanks to the various railroad and steamboat companies who have so liberally extended to us facilities of transportation, and to the daily press for their efficient aid in reporting the proceedings of this meeting.

On motion of J. P. Moore, of Mississippi, the following preamble and resolutions were adopted:—

*Whereas*, The contract system is contrary to medical ethics.

*Resolved*, That all contract physicians, as well as those guilty of bidding for practice, at less rates than those established by a majority of regular graduates of the same locality, be classed as irregular practitioners.

The following reports of the Sections followed:—

Section on Meteorology, Medical Topography, and Epidemics reported.—Paper accepted and referred to Committee on Publications.

Sections on Practical Medicine and Obstetrics reported and were accepted, and referred to Committee on Publications.

And the report on Training of Nurses was accepted, and the resolutions adopted.

Section on Medical Jurisprudence, Hygiene, and Physiology reported. Committee continued for next year. Report accepted, and referred to Committee on Publications.

Section on Surgery proposed that their report be received without formality and be referred to the Committee on Publications. Adopted.

After being read the report was accepted and ordered to be published.

Section on Pscychology, the same disposition.

The President appointed Dr. J. M. Toner a committee of one, at Washington, D. C., to assist the Librarian of Congress to keep the books of the Association.

On motion for adjournment, the President delivered an address, which was unanimously accepted and ordered to be published in the transactions of the Association.

GENTLEMEN:—Before I submit the motion just made, and which, when adopted, will practically close my official relations to this body, allow me to return you my most cordial and grateful thanks for the unvarying kindness which I have received at your hands. Whatever my future lot in life may be, the world holds no honors which to me can equal those conferred by you. The fraternal good-will which has so conspicuously marked your deliberations has been to me a matter of infinite satisfaction and pride, and will not be the least among the grateful memories which will gladden my heart as I may hereafter review the incidents of my official connection with you.

To win your judgment and approval, to hold up the dignity of fellowship, the usefulness of association, and the interest and prosperity of the profession at large have certainly occupied my most anxious thoughts since my elevation to this position; yet to cherish and promote the intimate and cordial relations of friendship between the individual members of this Association against all sectional distinctions or geographical lines, has also been among the chief objects of my ambition, and the earnest desires of my heart. Could I now believe that my efforts have contributed in the slightest degree to enlarging that harmony of sentiment and fraternal feeling which has been so apparent throughout this meeting, I should feel that I had commenced at least to make some return for the great honor and kindness received at your hands.

It now only remains for me, gentlemen, to again express to you my thanks, to wish you a safe return to your homes and labors, a happy reunion with your friends and families, and to pronounce that sad word over which the heart of friendship would fain linger, as I bid you an affectionate *farewell.*

W. O. BALDWIN,
*President A. M. A.*

The Convention adjourned to meet in Washington, D. C., second Tuesday in May, 1870.

